# Functional cure of hepatitis B requires silencing covalently closed circular and integrated hepatitis B virus DNA

**DOI:** 10.1172/JCI163175

**Published:** 2022-09-15

**Authors:** Marc G. Ghany, Anna S. Lok

**Affiliations:** 1 Liver Disease Branch, National Institute of Diabetes, Digestive and Kidney Diseases, NIH, Bethesda, Maryland, USA.; 2 Division of Gastroenterology and Hepatology, University of Michigan, Ann Arbor, Michigan, USA.

## Abstract

Chronic hepatitis B virus (HBV) infection remains a major global health problem. Hepatitis B surface antigen (HBsAg) loss has been accepted as the definition of a functional HBV cure. Recent studies found that while covalently closed circular DNA (cccDNA) is the predominant source of HBsAg in hepatitis B e antigen–positive (HBeAg-positive) patients, integrated HBV DNA (iDNA) is the main source in HBeAg-negative patients. Consequently, achieving a functional HBV cure will require not only silencing of cccDNA but also iDNA. Assays that distinguish the source of HBsAg are needed to evaluate emerging therapies. In this issue of the *JCI*, Grudda et al. developed a PCR-based assay that differentiated the source of HBsAg and explored the contributing sources of HBsAg in patients on nucleos(t)ide analog antivirals. These findings provide a tool for understanding the contribution of iDNA in HBV infection and may guide therapies toward a functional HBV cure.

## Hepatitis B virus life cycle

**Figure 1:**
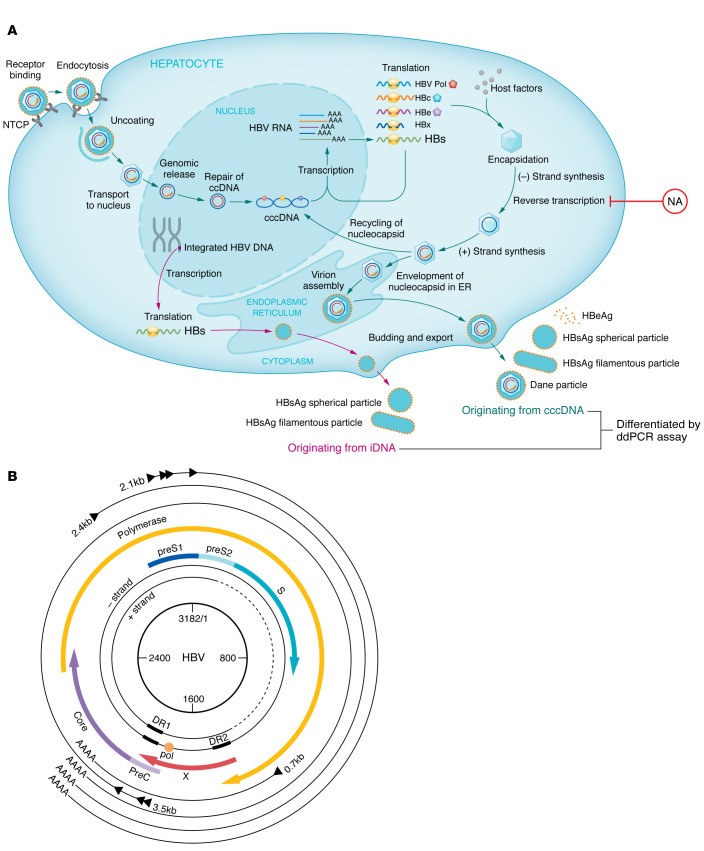
Illustration of the HBV life cycle and genomic organization. (**A**) HBV enters the hepatocyte via NTCP, a bile acid receptor. Following entry and uncoating, the nucleocapsid containing a partially double-stranded rcDNA translocates to the hepatocyte nucleus where host cellular enzymes convert the rcDNA into cccDNA. The cccDNA is a highly stable, supercoiled, episomal form of the viral DNA that serves as the template for transcription of all viral mRNAs, including the pgRNA. In the cytoplasm, the viral core protein self-assembles with the pgRNA and viral polymerase to form new nucleocapsids. Viral replication occurs within the nucleocapsid through reverse transcription of the pgRNA. The mature viral capsids containing rcDNA have two fates; they can be enveloped with the surface proteins in the endoplasmic reticulum and secreted from the infected cell as complete virions or they can be transported back to the nucleus to replenish the cccDNA pool. NAs inhibit genome replication by blocking reverse transcription of pgRNA, resulting in a rapid decrease in HBV DNA levels. Rarely, the minus-strand HBV DNA produces dslDNA instead of rcDNA. dslDNA can integrate into the host DNA as iDNA and can produce HBsAg. iDNA can continue to produce HBsAg even if cccDNA transcription is silenced and no longer produces HBsAg. Grudda et al. (15) developed a ddPCR assay that can differentiate HBsAg originating from cccDNA from that originating from iDNA. (**B**) The viral genome encodes four open reading frames that yield four mRNA transcripts with common 3′ ends. Notably, iDNAs lack the 3′ ends, which served as the basis for distinguishing the source of the HBsAg transcripts in the ddPCR assay. Four viral mRNAs — 3.5 kb pgRNA and 2.4 kb, 2.1 kb, and 0.7 kb mRNAs — yield precore (preC), core and polymerase, large, middle, and small HBsAg and X proteins, respectively. DR1, direct repeat 1; DR2, direct repeat 2.

Hepatitis B virus (HBV) is a small DNA virus that infects the liver. Its entry into hepatocytes is facilitated by the sodium-taurocholate–cotransporting polypeptide (NTCP), a bile acid receptor, which contributes to its hepatotropism (1). After entry, the nucleocapsid containing the partially double-stranded, relaxed circular DNA genome (rcDNA) of the complete virion is transported to the hepatocyte nucleus where host cellular enzymes repair the rcDNA to form the covalently closed circular DNA (cccDNA) (Figure 1A). The cccDNA is a highly stable, supercoiled, episomal form of the viral DNA. It serves as the template for transcription of all viral mRNAs, including the pregenomic RNA (pgRNA). In the cytoplasm, the viral core protein self-assembles with the pgRNA and viral polymerase to form nucleocapsids. Viral replication occurs within the nucleocapsid through reverse transcription of the pgRNA. The mature viral capsids containing rcDNA are then enveloped with the surface proteins in the endoplasmic reticulum and secreted from the infected cell as complete virions or transported back to the nucleus to replenish the cccDNA pool.

Subviral filamentous and spherical particles, comprising surface proteins but devoid of viral DNA, are produced in quantities that exceed complete virions by 1,000- to 100,000-fold. Rarely, during genome replication, there is defective primer translocation to the newly formed minus-strand DNA, leading to the formation of a double-stranded linear DNA (dslDNA) genome instead of the rcDNA genome. These dslDNA forms may integrate randomly into the host genome by utilizing sites of host cell DNA breaks (2). Recently, targeted long-read sequencing revealed that the full-length genome may be integrated but this integrated DNA (iDNA) does not support viral replication because it lacks the precore and core transcripts. Most of the transcribed integrants contain HBV promoter sequences that drive *S* transcript expression, resulting in production of hepatitis B surface antigen (HBsAg) (3).

## HBsAg loss defines a functional HBV cure

Globally, an estimated 296 million persons have chronic HBV infection, resulting in approximately 820,000 deaths annually predominantly from complications of cirrhosis and hepatocellular carcinoma (HCC) (4). Two classes of antiviral agents are approved for treatment of chronic hepatitis B, pegylated IFN-α and nucleos(t)ide analogs (NAs) (5). NAs are generally favored over pegylated IFN because they are more potent inhibitors of HBV replication, are administered orally, and have an excellent long-term safety profile. NAs inhibit HBV replication, primarily at the reverse transcription step. NAs do not have direct effects on cccDNA. Consequently, viral relapse is universal when NAs are stopped except for patients who have lost HBsAg (6). HBsAg loss is associated with durable suppression of viral replication, and a reduction in cirrhosis, HCC, and liver-related death (7–9). Thus, sustained HBsAg loss with undetectable HBV DNA after a finite course of therapy has been accepted as the goal for achieving a functional hepatitis B cure (10). However, HBsAg loss occurs rarely with NAs, less than 1% after 1 year and approximately 3% after 10 years (11). Therefore, most patients require long-term (many years or lifelong) NA therapy to maintain clinical benefit.

## Integrated HBV DNA as a source of HBsAg

Persistence of cccDNA is considered the Achilles’ heel of achieving a HBV cure, the reason why viral relapse occurs when NAs are stopped, and why HBV reactivation during immunosuppressive therapy can occur even after HBsAg is lost. It was believed that antiviral drugs that can eliminate cccDNA or completely silence cccDNA transcription would increase the rate of HBsAg loss. However, a recent study of siRNA therapy that targeted the common 3′ end of all HBV transcripts showed that while the treatment markedly reduced HBsAg levels in hepatitis B e antigen–positive (HBeAg-positive) patients, it had minimal effects on HBsAg levels in HBeAg-negative patients (12). Further analysis showed that HBV *S* transcripts from HBeAg-negative patients as well as chimpanzees frequently lacked the 3′ ends that were present in *S* transcripts from HBeAg-positive patients and chimpanzees. Altering the siRNA sequence to target a different region resulted in a decline in HBsAg levels in both HBeAg-positive and HBeAg-negative patients. These findings led to the revelation that the source of HBsAg in HBeAg-positive and HBeAg-negative patients may differ, being predominantly cccDNA in the former and iDNA in the latter. This notion of differential sources of HBsAg during different phases of chronic HBV infection has since been confirmed by other investigators (13, 14).

## An assay to differentiate the source of HBsAg

The knowledge that HBsAg may be derived from both cccDNA and iDNA presents a challenge for using HBsAg loss as a definition of functional cure and an endpoint in clinical trials of new therapies, as HBsAg may remain positive despite complete silencing and even elimination of cccDNA.

Current HBsAg assays cannot distinguish the source of HBsAg production. To address this issue, Grudda et al. devised a multiplex digital droplet PCR (ddPCR) assay that can differentiate the source of HBsAg (15). The assay takes advantage of the fact that iDNA-derived HBV *S* transcripts are chimeric with host DNA and lack the common 3′ ends that all cccDNA-derived transcripts share (Figure 1B). Thus, HBV *S* transcripts from iDNA would be detected by a mid-HBV but not 3′-HBV ddPCR assay, while HBV *S* transcripts from cccDNA would be detected by both mid- and 3′-HBV ddPCR assays. Utilizing paired liver tissues from a cohort of HIV/HBV-coinfected persons on chronic NA therapy, Grudda et al. used the assay to show that the source of HBsAg in HBeAg-positive patients was predominantly cccDNA, while that in HBeAg-negative patients was mainly iDNA. Furthermore, they showed that patients with greater decline in HBsAg levels between paired liver biopsies while on NA therapy was primarily due to a decrease in cccDNA-derived HBsAg, whereas patients with stable HBsAg levels had predominantly iDNA-derived HBsAg (15).

Grudda and colleagues next used laser capture microdissection to analyze viral transcription from individual hepatocytes from paired liver biopsies of three patients. One patient with marked HBsAg decline had a corresponding reduction in infected cells with transcriptionally active cccDNA, while the two patients without HBsAg decline had a stable or increased percentage of cells producing HBsAg from iDNA. The authors suggested their findings support a role for NA in transcriptional silencing of cccDNA but not a reduction in the number of infected cells. They further suggested that in patients whose HBsAg is mainly derived from iDNA, continuation of NA therapy will not achieve additional reduction in HBsAg levels and different therapies, targeting iDNA-derived HBV *S* transcripts or cells that produce them, will be needed (15). These findings conflict with other studies that have not reported an effect of NAs on cccDNA transcription or elimination (16). It is possible that a decline in cccDNA transcription after many years of NA therapy may be due to decreased nucleocapsid recycling and intracellular replenishment of cccDNA. Alternatively, not all hepatocytes targeted for microdissection harbored HBV infection, as no marker of infection was used for selecting HBV-infected hepatocytes (15).

## Implications for therapies aimed at functional HBV cure

The findings from Grudda et al. (15) and other studies showing that iDNA can be a predominant source of HBsAg in some patients, particularly those who are HBeAg-negative (12), and the low rates of HBsAg loss achieved in ongoing trials of emerging therapies that specifically target HBsAg (17), have raised the question whether HBsAg loss is a valid definition of functional HBV cure and if it is achievable. These findings imply that attainment of a high rate of HBsAg loss after a finite course of therapy would require not only sustained silencing of cccDNA transcription but also silencing of iDNA transcription or elimination of hepatocytes with iDNA. Silencing iDNA transcription may require gene editing with a potential risk of off-target effects or the creation of undesired mutations. Alternatively, silencing of iDNA transcription may be achieved via epigenetic modifications, which may be more specific and reversible (18). Attempting to eliminate hepatocytes that harbor iDNA might lead to hepatitis flares and liver failure, particularly since Grudda et al. and other investigators found that the percentage of hepatocytes harboring iDNA increases over time as patients transition from HBeAg-positive to HBeAg-negative (15). However, HBsAg loss does occur spontaneously in persons with chronic HBV infection and HBsAg loss can occur with current therapies: pegylated IFN-α and NA, albeit at low rates. HBsAg loss in these settings has generally been silent and not accompanied by hepatitis flares, and a few studies have demonstrated restoration of HBV-specific T cell responses after HBsAg loss (19). Understanding what leads to spontaneous or pegylated IFN– or NA-induced HBsAg loss will help in the design of therapies that can result in a functional HBV cure. Having blood-based assays that can differentiate the source of HBsAg will help confirm target engagement and monitor responses to therapy. Although the ddPCR assay developed by Grudda et al. relies on liver tissue, it can be a valuable tool in understanding the contribution of iDNA not only to HBsAg production but possibly also hepatocarcinogenesis, as iDNA is often detected in HBV-related HCC tissue (20).

## Version 1 09/15/2022

Electronic publication
